# The Association of Caries Increment Dynamics in Preschool Children with Risk Factors: The 3-Year Prospective Study

**DOI:** 10.3390/ijerph17207459

**Published:** 2020-10-13

**Authors:** Nela Pilbauerova, Eva Cermakova, Romana Koberova Ivancakova, Jakub Suchanek

**Affiliations:** 1 Department of Dentistry, Faculty of Medicine in Hradec Kralove, Charles University, and University Hospital Hradec Kralove, 500 05 Hradec Kralove, Czech Republic; nela.pilbauerova@lfhk.cuni.cz (N.P.); suchanekj@lfhk.cuni.cz (J.S.); 2 Department of Medical Biophysics, Faculty of Medicine in Hradec Kralove, Charles University, 500 03 Hradec Kralove, Czech Republic; cermakovae@lfhk.cuni.cz

**Keywords:** preschool children, caries increment, longitudinal study, prospective study

## Abstract

This prospective study monitored the dental status, the presence of plaque, and cariogenic microorganism levels of identical children over three years. The aim was to determine the dynamics of caries increment as well as the relationship between risk factors and caries prevalence. A total number of 125 children (72 boys and 53 girls) was included in the study, with an average age of 3.95 ± 0.06 years at the baseline. During the clinical examination at the nursery schools, the presence of dental plaque was recorded, and saliva samples were collected from the tongue of children for the DentoCult SM test providing easy detection of mutans streptococci from saliva samples. At baseline, 65.6% of the children had no caries, 4% had restored teeth with fillings or crowns or missing teeth due to caries, and 30.4% had at least one untreated caries. The percentages of intact teeth, restored or missing teeth, and untreated caries were 52.8%, 8.8%, 38.4% in the second year and 49.1%, 13.8%, and 31.1% in the third year. The dmft index value was 1.41 ± 0.24 in the first year, 2.29 ± 0.30 in the second year, and 2.33 ± 0.31 in the third year. There was a significant correlation between plaque presence and dt and dmft values (*p* < 0.05; the statistical analyses were performed using the Kolmogorov-Smirnov test). This 3-year longitudinal study highlighted the importance of examining both the oral hygiene and the level of cariogenic microorganisms when undertaking the evaluation of caries risk evaluation in preschool children.

## 1. Introduction

Tooth decay is the most common chronic disease and is particularly prevalent in children. Early childhood caries (ECC) is defined as a disease that affects the teeth of children from birth up to 71 months of age [1]. A well-established key measure of caries prevalence in dental epidemiology is the dmft index (where d—number of teeth with untreated dental caries, m—number of teeth extracted due to dental caries, f—number of teeth with caries treated with a filling or crown, t—teeth) [2]. The prevalence of dental caries of primary dentition in Czech preschool children has been repeatedly monitored for the last two decades [3]. A significant overall trend of declining caries prevalence, along with a drop in the mean number of teeth with untreated caries (dt), has been observed [4]. The average dmft index was 2.5 per child, with a dt value of 1.5 per child in both 3-year-old and 5-year-old children. The respective regression modelling of the caries risk indicators collected in the period 1994–2008 showed a significant declining mean dmft and mean dt value per child [3].

The most recent data reveal a 60% increase in caries-free children (aged 5), representing a 40% decline in caries prevalence compared to data collected in the 1990s [3]. Data collected in cross-sectional surveys on caries experience in regressive linear modelling can reflect the dynamics of oral health development in various age groups. However, these data are static data from the same age category but different children. The accurate picture of the caries prevalence dynamic can only be obtained by the repeated monitoring and investigation of identical children over time. Such a design of prospective, so-called incremental studies is very demanding in terms of organization and thus rarely seen in the scientific literature.

A similar situation is also evident from the monitoring of etiological factors, namely the amount of dental plaque, level of oral hygiene, level of cariogenic microorganisms, and nutrition habits (concerning the dentition conditions). The approximately similar degree of association between the plaque presence and level of cariogenic microorganism and caries prevalence has been demonstrated in many previous studies differing in the geographical, socioeconomic, and ethnic background of the cohorts studied [3,4,5].

The current view of caries prevalence in preschool and school children includes an evaluation of the measurable main risk factors to clarify their predictive value for oral health development [6].

Prospective studies in identical children are a methodological approach and can help clarify the effect of risk factors on caries progression and its dynamics. Such studies guarantee invariable caries evaluation and other observed factors [7,8]. We chose a prospective model to monitor the dental status, plaque, and cariogenic microorganism levels of preschool children and performed repeated examinations of the same children over three years. The study aimed to determine the dynamics of caries increment and gain a better understanding of the relationship between risk factors and caries prevalence. The null hypothesis of this study was that the caries increment is independent of the presence of plaque and cariogenic bacteria.

## 2. Materials and Methods

The guidelines for this 3-year longitudinal study were approved by the Ethics Committees of the Faculty of Medicine Charles University and University Hospital in Hradec Kralove (201803S14P). The legal guardians of the children had been adequately briefed before providing informed consent and allowing participation in the study.

### 2.1. Inclusion Criteria

All children aged 3–4 years were selected from nursery schools in Hradec Kralove, Czech Republic. All nursery schools were state, and private ones were excluded. We selected nursery schools in which a director approved the examination of children. At the time of the study, there were 49 state nursery schools in the city Hradec Kralove. The selection criteria were the number of children in each school, which means in particular section where the first examination was performed (first grade, children from three to four years of age). We have selected the nursery school with more than 20 children in the particular section. After this first selection, 20 nursery schools were left. The next criterion was the school director’s approval, after which 12 nursery schools were included in the study. The number of children in the study was 125 (from these 12 nursery schools), based on the written parental informed consent and the other mentioned inclusion criteria. A questionnaire survey was performed among the participant’s legal guardians to gather the required information about the respondents (personal information (i.e., gender, age), general health status, long-term used medication and allergies, personal oral hygiene habits, diet and daily fluoride intake). All children included in the study were free of systemic diseases and allergies. Legal guardians provided information in the questionnaire that they brushed child teeth twice a day using fluoride toothpaste. None of the included participants took any other fluoride supplements.

### 2.2. Clinical Cohort

We included 125 preschool children in our study at the baseline (72 boys and 53 girls, average age of 3.95 ± 0.06). Out of 125 children, 116 children were examined in the third year of the study (67 boys and 49 girls, average age of 5.08 ± 0.05). A sample calculation was necessary in order to find out that the size of the sample was sufficient. The sample size calculation was performed to find a minimum detectable difference in the dmft variable between the presence/absence of dental plaque for the sample size (*n* = 125 in the first and second years of the study, *n* = 116 in the third year of the study). This difference in values was found to be 1.7, which was considered as marginally significant. PASS 2019 software (PASS 2019 Power Analysis and Sample Size Software 2019, NCSS, LLC., Kaysville, UT, USA, ncss.com/software/pass) was used for calculations.

### 2.3. Examination

Teeth were clinically examined using a dental mirror, probe, and headlamp at the nursery schools. We followed the WHO methodology for caries detection limit of D3 lesions [9]. Examiners were trained to use the protocol approved for this study. Inter- and intra-examiner reliability was evaluated using kappa coefficients (0.82) before the beginning of the study during an examination of 20 three-year-old children regularly visiting the Department of Paediatric Dentistry, University Hospital in Hradec Kralove.

The data obtained from clinical examinations were recorded into standard forms using coding according to the above methodology. We also recorded the personal data (gender, date of birth, date of examination) of the children along with the examiner’s data.

The presence of dental plaque on buccal surfaces of upper frontal teeth was recorded using a periodontal probe during every year of the examinations. The plaque biofilm scrubbed off from the buccal cervical third (scores 2 and 3 according to the Silness and Löe plaque index [10]) of upper frontal teeth was recorded as positive (yes). During the clinical examination, we also collected saliva from children for the DentoCult SM test in each year of the examinations. The DentoCult SM test provides easy detection of mutans streptococci from saliva samples. After incubation, they are visible as light to dark blue, raised colonies on the rough area of the test strip. The classification classes are: 0: up to 10^4^ CFU (colony-forming unit); 1: 10^5^; 2: 10^5^–10^6^; 3: more than 10^6^ [11]. We performed the test according to the manufacturer’s instructions. Follow-up examinations were performed in the second and third years of the study, approximately one year from the baseline examination.

### 2.4. Evaluation of Caries and Caries Risk Indicators

We calculated the mean of dmft index and standard error (SEM) (d—number of teeth with untreated dental caries, m—number of teeth extracted due to dental caries, f—number of teeth with caries treated with a filling or crown, t—teeth) and dt index (mean number of teeth with untreated caries) per child. The plaque amount was evaluated in a binary manner (present/absent). Scores gained from the DentoCult SM tests were categorized according to reached score 0, 1—low risk, and 2, 3—high risk of caries prevalence.

### 2.5. Statistical Methods

The data are presented as the mean ± standard error of the mean (SEM). The two-sample t-test or Mann-Whitney or Kolmogorov-Smirnov test was used to determine statistically significant differences in age and caries indicators (dt, dmft). The dependence of both dt and dmft values/categories on gender was tested using Fisher’s exact test. The relationship between the DentoCult SM test scores and plaque presence of boys and girls were analyzed using the Chi-square test. The McNemar’s test of symmetry was used to analyze changes of the plaque presence or DentoCult SM test scores and caries indicators over the three years. The statistical difference in the caries increment was analyzed using the Wilcoxon’s test. The relationship between plaque presence and dmft or dt values was tested using the Kolmogorov-Smirnov test. A level of *p* < 0.05 was considered significant for all statistical tests.

## 3. Results

### 3.1. Clinical Cohort

In the first year of the study, we examined 125 children (72 boys and 53 girls) with an average age of 3.95 ± 0.06 years; the second year we saw 125 children (72 boys and 53 girls) with an average age of 4.67 ± 0.05 years; 116 children (67 boys and 49 girls) with an average age of 5.08 years ± 0.05 were examined in the third year of the study. The ages of the boys and girls did not differ significantly in the initial and second year, but they differed in the third year (*p* = 0.026) (illustrated in the Table 1). The intervals of follow-up examinations were approximately 11 months in the second year and ten months in the third year. The dt and dmft index values are depicted in Table 1. The mean number of teeth with untreated caries (dt index) was 0.96 ± 0.18 per child during the initial examination. In the second year of the study, the dt score was 1.28 ± 0.21 per child. In the third and final year, the dt index was 1.07 ± 0.17 per child. The average dmft index was 1.41 ± 0.24 per child for the first year, 2.29 ± 0.30 per child in the second year, and 2.33 ± 0.31 per child in the third year. We did not find significant differences between boys and girls in both dt and dmft indexes. The difference between boys and girls in dt values in the second-year examination was statistically non-significant (*p* = 0.098), as well as the difference between boys and girls in dmft value during the final examination (*p* = 0.074).

### 3.2. Examination

Table 2 illustrates the dentition status of children in the first-, second-, and third-year examinations. 

Statistical analyses were performed using the McNemar’s test at baseline, 65.6% of the children had no caries, 4% had restored teeth with fillings or crowns or missing teeth due to caries, and 30.4% had at least one untreated caries. The percentages of intact teeth, restored or missing teeth due to caries, and untreated caries were 52.8%, 8.8%, and 38.4% in the second year and 49.1%, 13.8%, and 31.1% in the third year, respectively. The dependence of both dt and dmft values on gender was not significant. However, the p values calculated in the second and third examinations were just outside levels of significance.

### 3.3. Plaque Presence and Cariogenic Microorganisms

The plaque presence on buccal surfaces of upper frontal teeth in children is shown in Table 3. Plaque was present on the buccal cervical third of upper frontal teeth in 38 children during the initial examination. These figures were 53 for the second-year examination and 24 for the final examination. The difference between boys and girls was not significant. However, the girls had better oral hygiene during each examination. There was a significant difference between the total number of children with a plaque in the first and second years of evaluations. This result is mainly due to significantly worse oral hygiene seen in boys (*p* < 0.001) in the second year of the study. However, we observed significantly improved oral hygiene in boys in the last year of evaluation (*p* < 0.001).

**Figure 1:**
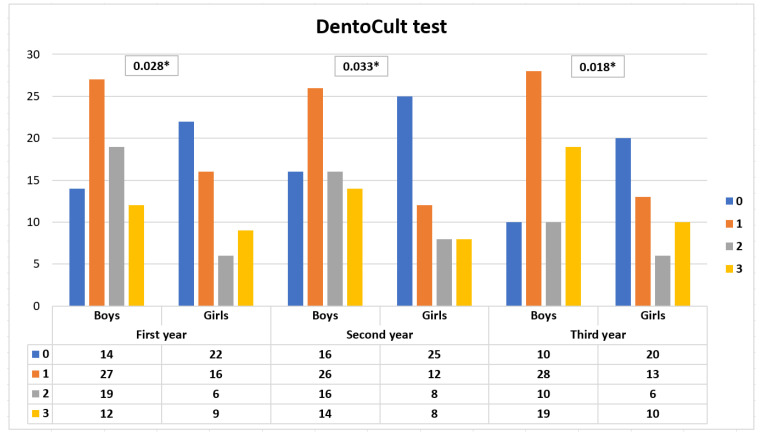
DentoCult SM test scores of boys and girls in the first, second, third year of the study. The statistical analyses were performed using the Chi-squared test. * *p* > 0.05.

DentoCult SM scores of boys and girls in the first, second, and third year of evaluations are depicted in Figure 1. 

Boys had significantly higher DentoCult SM scores in the first, second, and third examination than girls (*p* = 0.028, *p* = 0.033, *p* = 0.018). This result is mainly due to that girls had the DentoCult SM test score 0 more often than boys. Especially, girls had lower values (0 or 1) of the DentoCult SM test in the first year. The difference was not statistically significant (*p* = 0.091).

**Figure 2:**
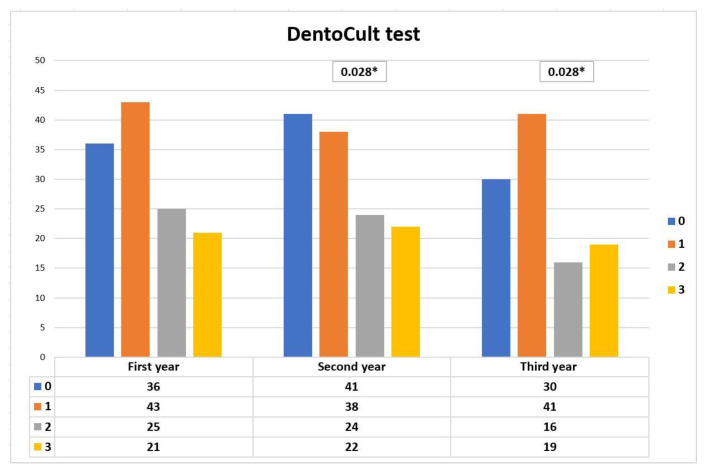
DentoCult SM test scores in the first, second, third year of the study. The statistical analyses were performed using the McNemar’s test, * *p* < 0.05.

During the study, we also studied the symmetry in the distribution of the DentoCult SM test scores over the years of evaluation. The differences in the DentoCult SM test score seen between the first and second year of the study, and between the first and third year of the study, were statistically significant. These results are mainly because of a decrease in the number of children having DentoCult SM test score 2 in the second and third years of evaluations (Figure 2).

### 3.4. Caries Increment

The monitoring of the caries increments (Δ dmft) is seen in Table 4. The average dmft index increments per child between the first and second and second and third evaluations were 0.88 ± 0.22 (boys 1.10 ± 0.27, girls 0.58 ± 0.36) and 0.19 ± 0.06 (boys 0.21 ± 0.08, girls 0.16 ± 0.10), respectively. The average dmft for boys and girls, all together, was significantly higher in the second and third years of the study compared to baseline. The dmft indexes for boys in the second year were significantly higher than for girls (1.10 vs. 0.58). The combined caries increment for boys and girls was significantly higher after the first compared to after the second year (0.88 vs. 0.19, respectively). This difference is caused mainly because of a lower caries increment seen in boys after the second year (0.21 ± 0.08).

### 3.5. Relationship between Plaque Presence and Caries Risk Indicators

A highly significant association between plaque and the carious tooth count (dt) or plaque and dmft values was found both in boys and girls during all examinations (Table 5).

### 3.6. Relationship between the Plaque Presence and DentoCult SM Test Score

The DentoCult SM test scores 0 and 1 (representing the low risk of caries) correlated with a lower number of children with plaque present on frontal teeth. Oppositely, scores 2 and 3 (signifying a high risk of caries) correlated with a higher number of children having plaque on frontal teeth. A significant relationship between plaque and DentoCult SM values in the first and second examinations (*p* < 0.001) was demonstrated. However, the same relationship was not seen in the third examination.

### 3.7. The DentoCult SM Test Score and Caries Indicators and Caries Increments

According to classification criteria, we also tested the hypothesis if the distribution of children with DentoCult SM test scores 0 or 1 differed in figures of dt, dmft, or caries increments (Δ dmft) from children with DentoCult test score 2 or 3. We did not find the distribution significantly different between the two categories (Table 6).

## 4. Discussion

The study aimed to determine the dynamics of caries increment and gain a better understanding of the relationship between risk factors and caries prevalence. There were no preventive dental interventions in the nursery schools from which the children were selected [12]. However, parents were told to brush child teeth regularly after meals with a fluoride-containing toothpaste. Children did not take any fluoride supplements.

The teeth of every child were examined using the dental mirror, headlamp, and probe. Cavitated carious lesions (D3) were the detection limit. The level of oral hygiene was evaluated by the presence or absence of plaque on the buccal cervical third of the upper front teeth. All the examinations were performed directly at the nursery schools, which made it somewhat tricky due to the lack of proper equipment with which to evaluate the initial non-cavitated caries/lesions and quantify plaque levels in more detail. However, the same methodology in preschool children had been used before by other authors [13,14,15]. The standardization of the re-examinations was ensured by the examiners/dentists.

The average age of the children at the beginning of the study was 3.95 years (approximately three years and eleven months). Out of 125 children, 116 children were examined in the third year of the study. The reasons for dropout were two: the child was no more visit the nursery school because of the residence change, or the child was not present at nursery school at the date of examination. The interval between the follow-up examinations was approximately 11 months. We extrapolated data for caries increment calculation to 12 months. At baseline, 65.6% of children had intact deciduous teeth. In comparison to a national survey in 2010, 44.9% of children in the approximately same age range had intact teeth with a dmft index of 2.9 [3].

We found no significant difference between boys and girls either in the average dmft index or in the mean number of teeth with untreated caries. The same results were published in a cross-sectional national survey in the Czech Republic in 2010 [3].

Out of 125 examined children, 65.6% had intact teeth during the initial examination. This figure was only 49.1% in the third year of examination. The restored teeth with filling of missing teeth due to caries, but no current decays, had 4.0% of children in the initial year and 13.8% of children in the final year of the examination. Unfortunately, more than one-third of children had at least one untreated caries in the first or third year of the examination.

Oral hygiene evaluated as plaque presence on the buccal cervical third of the upper front teeth was satisfactory in only a minority of examined children. Boys and girls did not differ significantly. However, we saw the better oral hygiene in girls, even though the difference was not statistically significant. Perhaps, girls (or their parents) showed a more significant interest in brushing teeth. Improvement in boy’s oral hygiene was mainly observed in the final year of the examination. Improvements in the age-related oral hygiene status in preschool children have been reported in other studies [16,17,18].

The number of children with the DentoCult SM test score 0 was 36 in the initial year, and the figure was 41 in the second year and 30 in the final year of examination. The changes in the DentoCult test SM score distribution seen between the second and third year of the study, and between the first and third year of the study, were statistically significant. Boys had a significantly higher DentoCult SM scores in the first, second, and third examination than girls (*p* = 0.028, *p* = 0.033, *p* = 0.018). This finding supported the better oral hygiene seen in girls in the initial, second, third year of the study.

We found a significant association between the presence of plaque and two caries indicators (the average value of the dmft and dt indexes) in the first, second, and third years of our longitudinal study. Many past prevalence studies have described the same positive association in preschool children [19,20,21]. Conversely, another study showed this association as non-significant [18]. Schröder et al. reported only an indication of this positive relationship [20]. Our outcome confirmed the importance of effective oral hygiene, proper brushing technique in preventing caries in deciduous teeth.

The distribution of children with DentoCult SM test scores 0 or 1 did not statistically differ in dt, dmft, or Δ dmft values from children with DentoCult SM test scores 2 or 3. This result is mainly because of the high deviations among children. Children, especially boys, had the worst level of oral hygiene in the second year. The dmft was 1.87 ± 0.33 for the group with the DentoCult SM test score 0 or 1 and 3.00 ± 0.59 for the group with the scores 2 or 3. The question is if the difference (1.13) is clinically significant, even though it is not statistically significant. We observed that the lower values of dt and dmft were associated with the DentoCult SM test lower scores in each year of this study.

Plaque presence and examinations using the DentoCult SM test are two critical markers of microbial caries risk. We should consider both when evaluating the potential risks of caries in preschool children. We confirmed a high correlation between plaque presence and DentoCult SM test values examined at baseline and the one-year follow-up. The results of the DentoCult SM test are thus greatly influenced by the level of personal oral hygiene. This conclusion, however, reduces the predictive value of the DentoCult SM test for the microbial origins of caries [22]. It is generally expected that chair-side microbial tests for cariogenic microorganism levels require an acceptable sensitivity and specificity when using them to predict potential higher carries risks [13]. Higher values of the DentoCult SM test are not associated with higher subsequent caries increments. Our findings confirmed the already known weakness of screening assessments of cariogenic streptococcal levels–the low sensitivity [23,24]. In other words, the DentoCult SM test is not enough on its own, but it is crucial in the evaluation of the individual’s risk of caries prevalence, especially in association with the plaque presence.

From a clinical point of view, the findings on the year-to-year increments are more important. A significant increase in caries was observed in boys and girls between the first and second, second and third, and first and third examinations. However, this increase was significantly higher after the first year than after the second year of the study. If we transferred this finding to the children’s age, it would mean that there is a higher risk of caries in children aged three to four-and-a-half than in children four-and-a-half to six. These observations should be reflected in the strategy of preventive and curative measures in preschool children.

There are some limitations to the present study. Some risk factors as dietary habits and the family’s social-economic status, being assessed in our previous studies [25,26], have not been implemented in this study. We are fully aware of this limitation, and further research is necessary for better understanding the relationship of caries risk factors and caries increment in preschool children.

## 5. Conclusions

Our three-year longitudinal study of caries increments in deciduous teeth with associated plaque presence and cariogenic microorganisms showed caries development dynamics. We partially rejected the null hypothesis. According to obtained data, caries increment is dependent on the plaque presence. However, the DentoCult SM test (bacteria presence) scores were not associated with higher subsequent caries increments. The study highlighted the importance of examining children’s oral hygiene (or efficiency in tooth brushing with the fluoride-containing toothpaste) and the level of cariogenic microorganisms when undertaking the evaluation of caries risk evaluation in preschool children. Our study pointed to the need for preventive measures regarding primary caries prevention, particularly for older preschool children.

## Figures and Tables

**Table 1:** The average age and gender of all children and their dmft index (d—number of teeth with untreated dental caries, m—number of teeth extracted due to dental caries, f—number of teeth with caries treated with a filling or crown, t—teeth).

Year of the Study		n	Age	d	m	f	dmft
1	boys	72	3.98 ± 0.08	0.86 ± 0.18	0.21 ± 0.13	0.26 ± 0.09	1.33 ± 0.28
	girls	53	3.90 ± 0.07	1.09 ± 0.33	0.17 ± 0.11	0.25 ± 0.09	1.51 ± 0.42
	total	125	3.95 ± 0.06	0.96 ± 0.18	0.19 ± 0.09	0.26 ± 0.07	1.41 ± 0.24
2	boys	72	4.73 ± 0.07	1.36 ± 0.26	0.42 ± 0.17	0.65 ± 0.16	2.43 ± 0.38
	girls	53	4.57 ± 0.06	1.17 ± 0.35	0.30 ± 0.12	0.62 ± 0.18	2.09 ± 0.50
	total	125	4.67 ± 0.05	1.28 ± 0.21	0.37 ± 0.11	0.64 ± 0.12	2.29 ± 0.30
3	boys	67	5.18 ± 0.07 *	1.18 ± 0.23	0.46 ± 0.18	0.93 ± 0.20	2.57 ± 0.39
	girls	49	4.94 ± 0.07 *	0.92 ± 0.27	0.29 ± 0.12	0.80 ± 0.23	2.00 ± 0.49
	total	116	5.08 ± 0.05	1.07 ± 0.17	0.39 ± 0.12	0.87 ± 0.15	2.33 ± 0.31

Data are presented as mean and SEM. Statistical analyses were performed using the t-test, Mann-Whitney or Kolmogorov-Smirnov test. * Bolded values represent the statistical significance level *p* < 0.05.

**Table 2:** Examination of the deciduous teeth.

Year of the Study		Boys		Girls		Total		Boys vs. Girls
		n	%	n	%	n	%	*p*-Values *
1.		72	100	53	100	125	100	
	dmft = 0	45	62.5	37	69.8	82	65.6	0.690
	dmft > 0; d = 0	3	4.2	2	3.8	5	4.0	
	dmft > 0; d > 0	24	33.3	14	26.4	38	30.4	
2.		72	100	53	100	125	100	
	dmft = 0	32	44.5	34	64.1	66	52.8	0.086
	dmft > 0; d = 0	7	9.7	4	7.6	11	8.8	
	dmft > 0; d > 0	33	45.8	15	28.3	48	38.4	
3.		67	100	49	100	116	100	0.090
	dmft = 0	27	40.3	30	61.2	57	49.1	
	dmft > 0; d = 0	11	16.4	5	10.2	16	13.8	
	dmft > 0; d > 0	29	43.3	14	28.6	43	31.1	

* Statistical analyses were performed using the Fisher’s exact test.

**Table 3:** The plaque presence and cariogenic microorganisms.

Year of the Study			Plaque Presence		*p*-Value			
		n	no	yes	Boys vs. Girls	1st vs. 2nd Year	2nd vs. 3rd Year	1st vs. 3rd Year
1	boys	72	51	21	0.727			
	girls	53	36	17				
	total	125	87	38				
2	boys	72	40	32	0.590	<0.001 *		
	girls	53	32	21		0.125		
	total	125	72	53		<0.001 *		
3	boys	67	54	13	0.689		0.002 *	0.189
	girls	49	38	11			0.115	0.481
	total	116	92	24			<0.001 *	0.108

Statistical analyses were performed using the McNemar’s test. * The bolted values represent the statistical significance level *p* < 0.05.

**Table 4:** Caries increases.

Examination		1.	2.	3.	1.–2.	2.–3.	1.–3.
dmft index		1.41 ± 0.24	2.29 ± 0.30	2.33 ± 0.31	<0.001 *	0.001 *	<0.001 *
Δ dmft	boys		1.10 ± 0.27	0.21 ± 0.08		<0.001 *	
	girls		0.58 ± 0.36	0.16 ± 0.10		0.335	
	total		0.88 ± 0.22	0.19 ± 0.06		0.002 *	

The statistical analyses were performed using the Wilcoxon’s test. * Bolded values represent the statistical significance level *p* < 0.05.

**Table 5:** The relationship between plaque presence and caries indicators.

Year of the Study	Caries Indicators	Plaque Presence		*p*-Values
		No	Yes	
1	dt	0.47 ± 0.11	2.08 ± 0.47	0.024 *
	dmft	0.70 ± 0.16	3.03 ± 0.63	0.024 *
2	dt	0.64 ± 0.19	2.15 ± 0.39	0.011 *
	dmft	1.38 ± 0.29	3.53 ± 0.56	0.027 *
3	dt	0.49 ± 0.11	3.29 ± 0.51	<0.01 *
	dmft	1.37 ± 0.26	6.00 ± 0.71	<0.01 *

The statistical analyses were performed using the Kolmogorov-Smirnov test. * Bolded values represent the statistical significance level *p* < 0.05.

**Table 6:** The correlation coefficients of the dmft values and DentoCult SM test values.

Year of the Study		DentoCult 0 + 1	DentoCult 2 + 3	*p*-Values *
1	dt	0.76 ± 0.19	1.30 ± 0.35	0.743
	dmft	1.09 ± 0.25	1.96 ± 0.48	0.726
2	dt	1.08 ± 0.24	1.63 ± 0.39	0.111
	dmft	1.87 ± 0.33	3.00 ± 0.59	0.694
3	dt	0.93 ± 0.22	1.29 ± 0.28	0.254
	dmft	2.00 ± 0.34	2.84 ± 0.57	0.683
1st–2nd	Δ dmft	0.72 ± 0.23	1.15 ± 0.44	0.999
2nd–3rd	Δ dmft	0.18 ± 0.08	0.20 ± 0.09	0.471
1st–3rd	Δ dmft	0.90 ± 0.20	1.02 ± 0.38	0.993

The statistical analyses were performed using the * Kolmogorov-Smirnov test.

## References

[1] American Academy of Pediatric Dentistry, American Academy of Pediatrics (2016). Policy on early childhood caries (ECC): Classifications, consequences, and preventive strategies. Pediatr. Dent..

[2] Larmas M. (2010). Has dental caries prevalence some connection with caries index values in adults?. Caries Res..

[3] Lenčová E., Pikhart H., Broukal Z. (2012). Early Childhood Caries Trends and Surveillance Shortcomings in the Czech Republic–Debate Article.

[4] Balkova S., Lencova E., Broukal Z. Trends in Oral Health of Children and Adolescents in the Czech Republic 1994–2006. Proceedings of the 13th EADPH Congress.

[5] Nair S., Kumar V.S., Krishnan R., Rajan P. (2017). A Comparative Evaluation of Bifidobacteria Levels in Early Childhood Caries and Severe Early Childhood Caries. J. Pharm. Bioallied Sci..

[6] Twetman S. (2015). Caries risk assessment in children: How accurate are we?. Eur. Arch. Paediatr. Dent..

[7] Slade G.D., Caplan D.J. (1999). Methodological issues in longitudinal epidemiologic studies of dental caries. Commun. Dent. Oral Epidemiol..

[8] Wong M., Lu H., Lo E. (2011). Caries increment over 2 years in preschool children: A life course approach. Int. J. Paediatr. Dent..

[9] Petersen P.E., Baez R. (2013). Oral Health Surveys: Basic Methods.

[10] Löe H. (1967). The Gingival Index, the Plaque Index and the Retention Index Systems. J. Periodont..

[11] Schlagenhauf U., Pommerencke K., Weiger R. (1995). Influence of toothbrushing, eating and smoking on Dentocult SM Strip mutans® test scores. Oral Microbiol. Immunol..

[12] Novotna M., Broukal Z. Oral Hygiene Practices and Diet Reported by Head-teachers in Czech Kindergartens: A Pilot Study. Proceedings of the 16th EADPH Congress.

[13] Meurman P., Pienihäkkinen K. (2010). Factors Associated with Caries Increment: A Longitudinal Study from 18 Months to 5 Years of Age. Caries Res..

[14] Seki M., Karakama F., Yamashita Y. (2003). Does a clinical evaluation of oral cleanliness correlate with caries incidence in preschool children? Findings from a cohort study. J. Oral Sci..

[15] Skeie M.S., Espelid I., Riordan P.J., Klock K.S. (2008). Caries increment in children aged 3-5 years in relation to parents’ dental attitudes: Oslo, Norway 2002 to 2004. Commun. Dent. Oral Epidemiol..

[16] Leroy R., Jara A., Martens L., Declerck D. (2011). Oral hygiene and gingival health in Flemish pre-school children. Commun. Dent. Health.

[17] Suma G., Das U.M., Ambika G. (2011). Jairanganath Oral Health Status of Normal Children and those Affiliated with Cardiac Diseases. J. Clin. Pediatr. Dent..

[18] Sutcliffe P., Murray J.J. (1996). Oral cleanliness and dental caries. The Prevention of Oral Disease.

[19] Kleemola-Kujala E., Räsänen L. (1982). Relationship of oral hygiene and sugar consumption to risk of caries in children. Commun. Dent. Oral Epidemiol..

[20] Schröder U., Granath L. (1983). Dietary habits and oral hygiene as predictors of caries in 3-year-old children. Commun. Dent. Oral Epidemiol..

[21] Sutcliffe P. (1977). Caries experience and oral cleanliness of 3- and 4-year-old children from deprived and non-deprived areas in Edinburgh, Scotland. Commun. Dent. Oral Epidemiol..

[22] Shi S., Deng Q., Hayashi Y., Yakushiji M., Machida Y., Liang Q. (2003). A follow-up study on three caries activity tests. J. Clin. Pediatr. Dent..

[23] Lenčová E., Broukal Z., Ivančaková R., Spížek J. (2010). Point-of-Care Salivary Microbial Tests for Detection of Cariogenic Species–Clinical Relevance Thereof–review. Folia Microbiol..

[24] Thenisch N., Bachmann L., Imfeld T., Minder T.L., Steurer J. (2006). Are Mutans Streptococci Detected in Preschool Children a Reliable Predictive Factor for Dental Caries Risk? A Systematic Review. Caries Res..

[25] Oganessian E., Koberova-Ivancakova R., Lencova E., Broukal Z. (2011). Alimentary fluoride intake in preschool children. BMC Public Health.

[26] Lencova E., Pikhart H., Broukal Z., Tsakos G. (2008). Relationship between parental locus of control and caries experience in preschool children–cross-sectional survey. BMC Public Health.

